# ‘Counselling is the only thing to do’: healthcare providers’ experience of Kangaroo Mother Care in Bangladesh – a qualitative study

**DOI:** 10.1136/bmjph-2024-001133

**Published:** 2024-11-28

**Authors:** Johanna Sjömar, Ylva Thernström Blomqvist, Taqbir Us Samad Talha, Syed Moshfiqur Rahman, Mats Målqvist

**Affiliations:** 1 Women and Childrens health, Uppsala Universitet, Uppsala, Sweden; 2 Uppsala Universitet, Uppsala, Sweden; 3 Maternal and Child Section, ICDDRB, Dhaka, Bangladesh; 4 Department of Women’s and Children’s Health, Uppsala University, Uppsala, Sweden

**Keywords:** Public Health, Education, Nursing, Education, Medical

## Abstract

**Aim:**

To explore healthcare providers’ (HCPs) experiences of facility-initiated kangaroo mother care (KMC) in Bangladesh.

**Methods:**

An explorative qualitative study based on 11 semistructured interviews with HCPs from 2 hospitals in Dhaka was conducted. Nurses, medical officers and paediatric consultants were representing the HCPs. Inductive, qualitative content analysis was used.

**Results:**

Supporting KMC needs to be a continuous process that requires a high level of commitment from healthcare staff. Optimal implementation is, however, challenged by structural conditions such as shortage of staff, separation of mother and child, and incomplete follow-up, which need to be addressed to support KMC.

**Conclusion:**

Findings from this formative research can help in designing interventions for scaling up KMC in Bangladesh by paying attention to the crucial role of HCPs and giving them training on the importance of continuous and repeated counselling to both mother and family. To enhance motivation, involving nurses in follow-up care is essential, alongside strengthening the health system for families living far away and tracking those not attending follow-up. Furthermore, after this study was conducted, the WHO updated its recommendations regarding KMC. This means that the current study, in combination with the WHO’s new recommendations, can be used for the development of new guidelines for KMC in clinical practice in Bangladesh.

**WHAT IS ALREADY KNOWN ON THIS TOPIC**

- High workload for healthcare providers could be a barrier to kangaroo mother care (KMC) initiation and provision.
- Motivating families with preterm babies to come for follow-ups can be a challenge for healthcare providers.

**WHAT THIS STUDY ADDS**

- Continuous counselling and committed staff to motivate parents and family members to be at the hospital and perform KMC are of utmost importance.
- Strengthening follow-up systems could act as a motivating factor for healthcare workers involved in KMC.

**HOW THIS STUDY MIGHT AFFECT RESEARCH, PRACTICE OR POLICY**

- A stronger emphasis on KMC as a continuous process not only for caregivers but also for healthcare staff is needed.
- More efforts to set up functioning follow-up systems of KMC continuation at home are needed.
- This study indicates the need to evaluate KMC practice in Bangladesh using new WHO recommendations.

## Introduction

 Preterm birth (born before gestational week 37), including low birthweight babies (less than 2500 g), is the leading cause of neonatal mortality. In 2020, 2.4 million neonatal deaths occurred among the 13.4 million babies born preterm worldwide in low-income and middle-income countries.^1 2^ Kangaroo mother care (KMC) is an intervention recommended by the WHO for babies with low birth weight, proven to prevent neonatal deaths and morbidity.^3^,^5^ KMC is defined as early, prolonged and continuous care of a newborn infant skin to skin with its mother or other caregiver, both in the hospital and after discharge, Ideally, exclusive breastfeeding/breastmilk feeding and proper follow-up are recommended.^3 5 6^ Furthermore, KMC should last as much and for as long as possible, and when skin-to-skin care cannot be continuous, it should be as frequent as possible.^6^ Based on evidence from recent studies, updated guidelines/recommendations from WHO have shifted from starting skin-to-skin care when the baby is considered medically stable to starting KMC immediately after birth to increase survival further.^1^

KMC is a complex intervention and there are challenges to its implementation and provision. Studies have pointed out several barriers within the health system, such as service delivery,^7^ the high burden of workload for health workers,^8^ adherence to following national guidelines due to an unsupportive environment and lack of staff.^9^ Further barriers described are limited readiness at the health facility to provide KMC, including lack of designated beds for KMC, proper documentation, lack of guidelines and trained and motivated health workers.^9 10^ On top of this, the lack of government support matters for the implementation.^11^ There are also barriers from the caregivers’ perspective that need to be considered, such as lack of support and care for their needs,^12^ family acceptance of the method, decision-making in the family, and costs and distance to the hospital.^13 14^ KMC can be implemented both in the facility and the community, mostly initiated in the hospital with a continuation at home after discharge. Different activities have been identified to be important for KMC implementation; following KMC coverage, identification of eligible babies for KMC, initiating and maintaining KMC in health facilities and supporting KMC after discharge. Also identifying challenges and adopting solutions to the specific context are important.^15 16^ To improve KMC implementation, an effort is needed both at the facility, regional, country and policy-maker levels as well as people acting as catalysts.^17 18^

### Rationale of the study

Sustainable Development Goal 3.2 refers to the neonatal mortality rate and aims to reduce preventable neonatal deaths to 12 deaths per 1000 live births or less by 2030 globally.^19^ In Bangladesh, (a lower-middle-income country), the neonatal mortality rate is 30 deaths per 1000 live births.^20^ A study investigated the causes of death for children under 5 years of age and showed that prematurity and low birth weight represent 11% of neonatal deaths in Bangladesh.^21^ The Government of Bangladesh adopted KMC as one of the priority interventions to be scaled up nationally in Bangladesh’s Every Newborn Action Plan^22^ to address preterm birth. A national guideline was finalised in 2015 for KMC and this guideline was updated in 2021. The first 4 years of KMC implementation in Bangladesh, from 2016 to 2020, are described by Ehtesham *et al*^23^ and they found that the enrolment of eligible babies was around 20%. They also found a reduction in mortality among babies cared for by KMC and that the follow-up rates were low. Implementation research shows the importance of finding contextual adoption and solutions for a specific intervention. It is, therefore, important to investigate the experiences of KMC to understand how to adapt it to the local context. This is identified as a priority research question in Bangladesh.^24^

### Aim

The aim of the study was to explore healthcare providers’ (HCPs) experiences of facility-initiated KMC in Bangladesh.

## Material and method

### Study design

This is an explorative qualitative study based on semistructured interviews with HCPs.^25^

### Setting

The study took place in two hospitals in Dhaka, Bangladesh, where KMC service is provided. One of the hospitals, Mohammadpur Fertility Training Center (hospital A), is a secondary-level public hospital that serves as a referral centre for maternal and child care services beyond basic primary care. There is a KMC corner with three beds dedicated to KMC at the time of the study. The other hospital, the Institute of Child and Mother Health (hospital B), is a tertiary-level public hospital that serves as a referral centre, receiving patients from lower-level healthcare facilities and focusing on complex cases related to maternal and child health. There are 15 beds dedicated to KMC in a separate KMC ward. The selection of hospitals was done purposively to capture a broad range of patients from different social backgrounds. At the time of data collection, hospital A did not have a neonatal intensive care unit and could not give breathing support and patients were referred if needed. Hospital B had a neonatal intensive care unit and could give breathing support and there was no need to refer patients as they could handle most of the cases in-house, except cardiac surgery. None of the hospitals had a systematic and structured use of donor breast milk. More detailed information about the hospitals is described elsewhere.^12^

### Participants

In total, 11 HCPs participated in the study from the 2 hospitals. Purposive sampling was used for the participants willing to participate in the interviews. The participants were either proposed by the manager or senior nurse to be interviewed and should be available during the data collection. The HCPs had different positions; nurses, medical officers and paediatric consultants. An eligibility criterion was to have at least 1 year of working experience with KMC.

### Data collection

The data collection was performed in August 2019. Semistructured interviews based on an interview guide were conducted (see online supplemental material ‘Interview_ guidelines_ HCPs_ English’). The interviews took place at the hospital, either in a separate room or somewhere in the KMC ward, where it was assessed that the interview could be conducted without disturbances. Privacy was secured by making sure no other staff was present during the interviews in the KMC ward and no overhearing was possible. The interview guide was developed by JS together with the input of the researchers from icddr,b, and from the Department of Women’s and Children’s Health at Uppsala University. The interview guide was pilot-tested with one interview, and this was included in the study. No changes were made to the interview guide and the guide was assessed to be suitable for addressing the purpose of the study. The data collection team consisted of (JS), a female paediatric nurse with a Master’s in Public Health and previous training and experience in qualitative methodology and performing interviews, a male anthropologist (TUST) and a female interviewer, both native Bengali speakers employed at icddr,b with training and experience of performing interviews. The data collection team introduced themselves and explained the aim of the study. The interviews started after participants gave their written consent to participate. Everyone who was asked chose to participate. The focus of the questions was to explore the HCPs’ experiences regarding KMC and what could be facilitators and barriers in the work with KMC. The interviews were performed face to face in their native language Bengali, one interview was done in English according to the participant’s choice. Field notes were taken by JS during the interviews. Discussion of the findings from the interviews was done after each interview by the data collection team and data saturation was discussed and considered as achieved during the last interviews as they gave similar content. The interviews lasted between 36 and 63 min (average 48 min) and were audio recorded, transcribed verbatim and translated into English. One participant was reinterviewed due to disturbing noise that was discovered during the transcription.

### Data analysis

Inductive qualitative content analysis was used as a method for analysing the interviews.^26 27^ JS and YTB read and re-read the transcripts to familiarise themselves with data and identify meaning units. Afterwards, each meaning unit was manually coded and sorted into possible subcategories which were merged into categories describing the content of the interviews by JS and YTB. Discussion within the research team applying a back-and-forth process continued until a consensus was reached for the subcategories and categories. Quotes were extracted from transcribed interviews and included in the text to illustrate the findings. The findings were not returned to the participants for comments or feedback. Word and Excel software was used to sort the data and meaning units from the text were moved from Word to Excel by ‘Add-in’.^28^

## Results

In total, 11 HCPs participated in the study, 5 HCPs from hospital A and 6 HCPs from hospital B. Two were paediatric consultants, three were medical officers and six were nurses. All the different professions represented both hospitals. Three participants were male, eight were female and all were between 25 and 54 years old. All had received at least 3–5 days of training on KMC. The data analysis resulted in the following three categories based on nine subcategories as outlined in table 1.

**Table 1:** Healthcare providers’ experiences of facility-initiated KMC in Bangladesh are organised into categories and subcategories

Categories	Subcategories
A continuous process is needed	Repeated counselling and demonstration facilitate acceptance
	Useful to invest time in motivational work
	Being attentive to changing needs
Staff commitment is decisive	Training and supervision make staff better
	Low intensive care requires endurance
	A method to believe in and a source of pride
Supporting KMC is challenged by structural conditions	Lack of facility readiness
	Clinical routines promote initial separation
	Contextual factors affect breastfeeding and breastmilk routines
	Pull factors to a non-conducive environment at home
	Incomplete follow-up routines

KMCkangaroo mother care

### A continuous process is needed

Early initiated and repeated counselling and demonstration by the HCPs to the baby’s parents and other family members were perceived to facilitate acceptance of KMC. HCPs stressed that this was a continuous process that required time investment, repetition and staff to be attentive to the changing needs of the KMC provider.

### Repeated counselling and demonstration facilitate acceptance

The HCPs said the counselling started immediately when the baby arrived at KMC ward/corner. Since KMC was a new concept for the mother and family, they said it was a gradual process facilitated by counselling. Before initiating skin-to-skin care, other family members, including the father, grandparents, sisters and aunts, were considered potential coproviders of KMC and included in the counselling session, as the decision to practice KMC was made collectively. All caregivers available in the hospital received counselling. Using a variety of tools, such as leaflets, pictures, television (TV), hands-on demonstrations and occasionally real baby demonstrations, was necessary for effective counselling according to the HCPs. They said the goal was to provide the mother with self-sufficiency and confidence in her ability to practice KMC. The counselling covered the benefits, necessity, procedure, time, length, home continuation and economic advantages of KMC over incubators. To protect the baby, they also emphasised hygiene issues. The HCPs said that the counselling was conducted individually and in group settings, where mothers and other family members were receptive and actively involved. During counselling, the HCPs stressed their emphasis on the mother’s and family’s needs and carefully addressed any questions that emerged. The process of counselling necessitated the repetition of information and demonstration, which demanded time and effort from HCPs. Although they recognised its importance, they also found it challenging and time-consuming.

Counseling is the only thing to do, listen to their problems, motivate, talk about benefits, show them records of temperature, heart rate, glucose…. (Healthcare provider 3)

### Useful to invest time in motivational work

To motivate the family, HCPs saw the need to emphasise the benefits of KMC, such as promoting the bonding between mother and baby and the mental satisfaction of the mother. The HCPs said it was useful to invest time in motivational work, which is a part of their job since it takes time for the mothers/families to understand what KMC is and why it is important. The HCPs said they were sometimes accused by the mothers or the family and they expected the baby should be an incubator. Illiteracy was brought up as a hindrance to starting KMC since the awareness of KMC was not there before coming to the hospital. If awareness of KMC was in place in the community and at the antenatal care that could facilitate the acceptance. The motivational work was also considered important for motivating the family to come for the follow-up.

Initially mothers were unwilling to perform KMC, and they were not sure about its effectiveness. Sometimes they give consent initially to give KMC, but later we saw they kept babies beside. All these happen due to illiteracy and ignorance…There are other superstitions too. In case of vaccination also, mothers were unwilling at the beginning, but now they come with babies spontaneously. (Healthcare provider 4)

### Being attentive to caregivers’ changing needs

The HCPs expressed that the issue of mothers’ comfort has to be taken seriously and their needs have to be considered as they could change over time. The HCPs talked about how mothers sometimes complained that it was too cold or too hot in the KMC-ward/corner and the baby was sweating and therefore uncomfortable, especially during the summer season. Sometimes when the air-conditioners were used it was perceived as too cold and the HCPs said the mothers were worried about the risk of hypothermia for their babies.

There are many who sweat a lot, they usually complain on that note. (Healthcare provider 6)

The HCPs tried to make it convenient for the mothers by drying the baby and changing the binder; however, if the number of patients was high, the binders were not washed between patients due to time constraints. Pain after a caesarean section was mentioned as another challenge for the mothers. The HCPs felt they considered privacy for the caregivers/mothers but that it was not enough. The HCPs emphasised that they tried to ensure privacy. In the KMC corner, visitors were allowed but there was a curtain separating the beds. No male visitors could enter the KMC ward.

### Staff commitment is decisive

Committed HCPs, training and supervision were expressed to be essential. KMC was explained as a method they trusted in and felt proud of, even though some expressed disinterest in working at the KMC ward and feelings of boredom among HCPs and caregivers were perceived as common.

### A method to believe in and a source of pride

The HCPs emphasised they had a positive attitude towards KMC. In the interviews, it came up that teamwork was needed among the HCPs, and that they have to be compassionate and dedicated to their work and believe in KMC. The HCPs also expressed positive feelings about KMC and that they felt joy and pride in providing this service when seeing patients’ improvements. They expressed that KMC was universal and should be for everyone regardless of race and gender. They witnessed that mothers are happy when their baby improves and would remain worried if the baby were separated in an incubator, as opposed to KMC where the mother can touch the baby and also breastfeed.

I felt glad when I could set a tiny baby on mother’s chest. Previously, we used incubators, but now we do not need that, because mother’s chest is an incubator itself. I feel good when I see a baby with less weight is getting better, it is not getting infected or it is being free from the respiratory problem. I feel good. (Healthcare provider 7)

### Training and supervision make staff better

All HCPs said they had received training about KMC and were positive towards training. They emphasised that training and supervision could help to motivate them to work in the KMC ward/corner. Some asked for more practical training on the different parts of KMC, like hands-on training or refresher training. At the same time, it was also mentioned that it was not always the HCPs could work according to what they had learnt in the training due to lack of manpower. They said supervision was considered very important and that it was done regularly, both by senior nurses and doctors/medical consultants as well as people from outside like the Ministry of Health, Save the Children or UNICEF.

It is definitely necessary. For the baby’s mother and the babies. Moreover, because we are trying to make sure we are doing the right thing. Supervision makes us even better. It also enables us to do good work with our highest efforts. (Healthcare provider 2)

### Low intensive care requires endurance

It was expressed that, at times, there was resistance among the HCPs to being in the KMC ward. Feelings of being bored were mentioned and the HCPs didn’t want to be assigned to the KMC-ward, especially in the beginning when KMC started. The HCPs mentioned that even if they feel bored, they go and monitor the babies. The HCPs thought that also the mothers could feel bored in the KMC ward/corner and that it would be good to have options for some entertainment such as TV or a library with books in the KMC ward.

Initially nurses also were not coming here willingly, but eventually they are also having satisfaction. (Healthcare provider 4)

### Supporting KMC is challenged by structural conditions

Lack of staff and equipment were mentioned to challenge KMC. Clinical routines were said to be barriers to initiating KMC and to proper follow-up after discharge. Financial barriers make mothers disinterested in staying in the healthcare facility and they leave the hospital before completing the KMC service. Duties at home, the baby’s well-being and distance to the hospital were told to affect the parents’ or family member’s willingness to perform KMC both at home and in the hospital and to come for follow-up.

### Lack of facility readiness

The HCPs witnessed a shortage of staff, as well shortage of beds and chairs for the KMC care. There was a lack of equipment like medicines, pulse oximeters and cannulas and the HCPs said they often had to leave the caregivers alone with the baby while they went out from the KMC-ward to bring the equipment. They suggested having dedicated staff for KMC patients instead of rotating staff. Staffing should be adjusted based on the need to ensure efficient work. The HCPs suggested creating a separate KMC ward instead of the KMC corner.

If we had the manpower, we could have given KMC care better. And there is insufficient space to increase the number of beds that more KMC mothers can avail for KMC services. (Healthcare provider 2)

The HCPs said that binders were given in the hospital but not in sufficient amounts and sometimes binders could not be washed between patients as it took time to wash and dry the binders. The HCPs said it was important to show that binders could be made from different materials and could be made at home and sometimes parents asked for samples so they could replicate by themselves. It was brought up that the handwashing facilities for the mothers could be improved as well as facilities for relatives. Air conditioning was said to be good to have at the KMC ward/corner but could also create a dilemma for the mothers if they have different opinions.

### Clinical routines promote initial separation

The HCPs witnessed a common initial separation between the mother and her baby, either due to the mother’s condition, caesarean section or pain after caesarean section or because the baby’s condition was not stable, and therefore, was admitted to the neonatal intensive care unit where the mother was only allowed to visit only for breast feeding. Sometimes babies needed to be referred to another hospital or back to the neonatal intensive care unit which prolonged the separation. The HCPs expressed they knew the criteria for skin-to-skin care, and this could not start until the baby was stable according to the national guidelines and all emphasised they follow strictly these guidelines.

We cannot provide KMC to all babies with low weight, because in that case, we have to stabilize the baby. All the babies who are in a stable state, those babies can be provided with KMC. (Healthcare provider 10)

### Contextual factors affect breastfeeding and breastmilk routines

According to the HCPs, babies not able to suck or in bad condition received intravenous fluids the first few days after birth or were given expressed breastmilk by oral-gastric tube or by cup before breastfeeding started. The HCPs said that breastfeeding counselling included how the baby should be positioned and that the baby should be breastfed every 2 hours and on-demand and that sometimes even the father got counselling about breast feeding. They explained that if the mother did not have enough breast milk, she was counselled about the need for her to eat and drink to produce milk. It was mentioned that to keep privacy during breastfeeding and expressing breastmilk, curtains or a movable screen were used to ensure privacy for the mother. The HCPs said the milk was expressed by hand and given at once, there was no habit of storing breastmilk in the fridge. During expressing breastmilk no males were allowed.

We don't face barrier that much. Small baby won't suck the breastmilk that well. When it gradually improves, we give counseling. We see if the baby is gaining weight. (Health care provider 5)

Some cultural and gender issues were brought up concerning donated breast milk. It was described that breastmilk from a Hindu mother could not be given to a Muslim and vice versa. The same was if the baby had a different gender, a boy could not have donated breast milk from a mother to a baby girl. It was also brought up that breast feeding could be tiring and hard for the mothers, especially when having twins or triplets.

### Pull factors to a non-conducive environment at home

Duties at home and the fact that someone from the family needed to stay with the mother in the hospital were factors that affected the hospital stay according to the HCPs.

Difficulties to stay in KMC due to costs were another factor that was mentioned. If there was a need, families were sent to social welfare for financial support to be able to stay longer in the hospital. The absence of a family member accompanying the mother in the hospital and other children to take care of were reasons for the mother to go back home mentioned by the HCPs. They said it is hard for the mother to perform KMC at home where she can feel pressure from the family to do household chores. In the hospital, it is a protected time for KMC, and the mother can get comfort. The HCPs told the family that they need to share responsibilities at home so that someone can give KMC for the baby.

In this case only telling this to the mother (to continue KMC at home) will not help, their family should be aware of it too. Because mothers might want to provide KMC, but others don't. In that case it is quite impossible for a mother to provide this care alone. In that case, we explain to the father and baby’s relatives what their duty is. Without their support it will be impossible for the mothers to recover this baby. Then they agree and it helps the mother. (Healthcare provider 6)

### Incomplete follow-up

The HCPs said they encouraged the caregivers to continue KMC at home with assistance from family members for household tasks. HPCs explained the concept of ‘resting 1 month’ after birth, which aids the mothers in providing KMC during the early weeks at home. The family was instructed to return to the hospital for a follow-up after 7 days and then weekly for four follow-ups. Nevertheless, not everyone attended all the subsequent appointments. HCPs suggested that the family’s comprehension of the importance of follow-ups may have been lacking and may have skipped them if the baby’s condition was satisfactory. Additionally, due to distance and travel costs, follow-up was hindered. Doctors used SMS or phone calls to remind families about follow-ups, which were occasionally missed due to a heavy workload. Meeting the baby during follow-up was considered a potential motivator by one nurse and to involve nurses in the follow-up was a suggestion. Another suggestion that was raised was to find other solutions for the follow-up if there was a long distance for the family to come to the hospital; a nearby health facility could take care of follow-up babies and that information should be linked between these centres.

We do not have that facility (nurses involved in the follow-up), but it would have improved the quality of service or we could have feedback on whether the babies are getting better or not. (Health care provider 9)

## Discussion

Results from this exploratory study of HCPs’ perspectives of facility-initiated KMC in Bangladesh indicate that KMC needs to be a continuous process that requires a high level of commitment from HCPs. Furthermore, it highlights that optimal implementation is challenged by structural conditions such as shortage of staff, separation of mother and child and incomplete follow-up that need to be addressed to support KMC.

The continuous process emphasised by the HCPs shows the importance of involving and counselling the parents and family members repeatedly. Previously, a study from Bangladesh indicated that counselling is often incomplete and not done regularly.^9^ As the mother cannot decide by herself, the available family members need to be counselled to accept to start with KMC. In Bangladesh, KMC is offered to the families and they can say yes or no to KMC, whereas in other countries KMC is a part of standard care. Including KMC care in Bangladesh as standard care could potentially increase the uptake of KMC.

We have previously described that the conditions for the caregivers to provide KMC exist in this setting,^12^ but it is important to meet the mothers’ needs in the forms of practical support for caring for their babies skin to skin, caring for their privacy and to their need of painkillers after caesarean section. This is in line with a WHO position paper on KMC from 2023,^29^ which highlights the importance of addressing the mothers’ and caregivers’ needs of comfort, as they have a central role in the care in KMC.

Previous research has shown that HCPs’ belief in the importance of KMC matters, and that persons passionate about KMC are needed for successful implementation, both in low-middle-income and high-income settings.^30^ Committed staff and a positive attitude towards KMC were highlighted as enablers also in our study. One way to ensure this, which was pointed out as an important issue, was training and regular supervision for the HCPs, both external and internal. Similar findings have been described in a study from Indonesia^31^ and a systematic review from sub-Saharan Africa.^14^ Furthermore, teamwork among HCPs, such as nurses and doctors, is needed for the support of KMC^30^ as also highlighted in our study.

However, we found contrasting views about KMC. Some HCPs were very positive toward KMC, while some saw the method as low-endurance care and said they were not always willing to be in the KMC ward since they felt bored. On the other hand, other HCPs said they were proud of the method when they saw the benefits it brought to the babies. This shows that attitude matters, which has also been reported in the literature.^14^

The participants in our study emphasised that they were strictly following the existing national guidelines for providing KMC only for stabilised children. Furthermore, the HCPs said that babies and mothers often are separated when the babies need intensive care, and caring for mother and child as a couplet is not in place during these circumstances. Recently updated recommendations from WHO have, however, shifted from starting skin-to-skin care when the baby is considered medically stable to starting KMC immediately after birth to increase survival.^32^ Even older studies have shown that babies could be stabilised better if in KMC position immediately after birth.^33 34^ Since there are new recommendations about KMC from WHO in 2022, there is a need to evaluate KMC practice in Bangladesh using the new WHO recommendations. The new WHO guidelines point out that this requires changes in the health system building blocks.^35^ A study looking at barriers and enablers to KMC before stability found that HCPs were concerned about the safety of introducing KMC to non-stable babies and that mothers are unavailable for this if in pain after caesarean section or other medical concerns.^36^ The result from our study witness of a common initial separation of mother and baby. Research has shown that early onset of skin-to-skin care leads to more skin-to-skin care during the care period.^37^ Therefore, families need to be empowered and supported to take their central places as providers of care for their preterm and LWB infants and not be separated.^38^ This is in line with Family Centred Care^39^ and the new KMC position paper from WHO that emphasises caring for mother and child together and having a conducive environment supporting this.^29^ A study from Ethiopia highlights the need for a physical space to be able to care for the mother and baby together.^40^ A study aiming for higher KMC coverage^15^ emphasises the importance of not separating the mother and baby, and to aim for zero separation, allowing the mothers to have a central role in caring for their babies in the hospital and providing an integrated care.

A final finding worth highlighting is the role of follow-up and support for continued KMC after discharge. Our result shows that the mothers feel pressured to return home with their babies earlier than the doctor’s recommendation due to different pull factors. HCPs perceive follow-up to be incomplete, and that it is something they are not all involved in. Feedback from follow-up was stressed as important for the HCPs and that it could increase their motivation and it was suggested to involve nurses from the KMC ward in the follow-up care. To our knowledge, this has not been described anywhere else.

### Strengths and limitations

To increase trustworthiness we have attempted to provide a detailed description of the context, data collection and how the analysis was performed and enhance the possibility of transferability to other similar settings. The interviews were performed in the participants’ native language and represented different professions to capture a broad view of their experiences, which further strengthened our study. The transcripts and translations were checked towards the audio files by native Bangla-speaking persons from our research team to try to avoid some nuances and words getting lost during transcription and translation. There might have been biasing with the selection of the participants as the managers or senior nurse subconsciously or consciously might have selected HCPs’ in favour of KMC. Social desirability bias could provide a limitation of the study, as the HCPs might have said things, they thought we wanted to hear and thus not reflect reality. There might also be some information they did not want to be shared, and therefore, not mentioned in the interviews. We attempted to reduce this risk by trying to keep a friendly atmosphere and give a detailed description of the aim and interest of the study and data collection team. The interviews were rich in data, the participants were well spoken and we found similar things brought up in the interviews, and therefore, found the data to be saturated. Another strength of this study is that the research team consisted of a diverse group of paediatric nurses, a paediatrician, a professor in Global Health, and an anthropologist with different experiences within the field of KMC, nursing, qualitative methodology, implementation and research in Bangladesh and similar settings.

## Conclusion

The findings from this formative research can help design interventions for scaling up KMC in Bangladesh by paying attention to the crucial role of HCPs and giving them training on the importance of continuous and repeated counselling to both mother and family. To increase their motivation, there is a need to involve nurses in the follow-up, while at the same time strengthening the follow-up system by finding solutions if the family lives far away and tracing the ones not coming for follow-up. Furthermore, after this study was conducted, the WHO updated its recommendations regarding KMC. This means that the current study, in combination with the WHO’s new recommendations, can be used for the development of new guidelines for KMC in clinical practice in Bangladesh.

## supplementary material

10.1136/bmjph-2024-001133 online supplemental file 1

## Data Availability

Data are available on reasonable request.
