# Supplementary material for: ‘Counselling is the only thing to do’: healthcare providers’ experience of Kangaroo Mother Care in Bangladesh – a qualitative study
Source: BMJ Public Health. 2024 Nov 28;2(2):e001133. doi: 10.1136/bmjph-2024-001133 (PMC11816417; doi:10.1136/bmjph-2024-001133)
Supplement: online supplemental file 1 [file bmjph-2-2-s001.pdf]

Supplementary material

Interview guide for questions to healthcare providers-English

Inform about the aim of the interview

The aim with the interview is to explore and describe the KMC providers experience of the Kangaroo Mother Care with a focus on enablers and barriers

- Introduction of principal investigator and interviewer
- Inform about confidentiality and volontary participation
- Signing of consent form
- Background information: see a different form

Supplementary questions/in-depth questions

- Tell me more…
- How was it when…
- How did you percieve…
- Can you tell me more about how you felt…

Initial question

What do you think of Kangaroo Mother Care? How do you define KMC? What are the benefits of KMC?

Specific questions

1. How do you **motivate** the caregivers to perform KMC? How is the process of that motivation?
2. How do you **support** the parents/caregivers to perform:
    - skin to skin (teaching the caregivers about position of skin to skin care, how to sit during skin to care, timing of skin to care, initiation and duration of skin to skin care, help given when the caregivers goes to toilet, prepare food
    - breastfeed/ giving breastmilk? (teaching the caregivers about the position and attachment of breastfeeding, giving breastmilk, or extracting breastmilk, teaching the mother to provide cupfeeding if baby don´t suck, initiation and duration of breastfeeding or cup/ nasogastric tube
3. What are the existing facilities to provide skin to skin care (binder, fan, AC, KMC bed, mothers waiting area?
4. What are the existing facilities for expressing breastmilk? (privacy, pump, storage of breastmilk, spoon or cup feeding, nasogastric tube).
5. Do you have a monitor checklist to track the timing of the skin to skin, for how long the mother providing skin to skin care, how many times the mothers providing breastmilk for the baby, how do you document and how is the reportmechanism for that? If yes, what are the problems in managing the checklist, monitoring and reporting?
6. **What can support/ facilitate** KMC to work? (leadership, managament). Do you have any external supervision who is responsible for the KMC. What do they do during the supervision? Do you think this supervision is beneficial of improving the quality of KMC of your facility?
7. **What can facilitate** the implementation of KMC? (training, refreshertraining, supervision, availability of KMC logisitics and supplies, documentation. If not available what are the reason behind that and how can we strengthen the supervision or monitoring mechanism.
8. What motivates you as healthcare staff to work with KMC/ to continue working with KMC?
9. **What can be barriers/ hindrance** for the KMC to work? Barriers for you as healthcare staff to support parents/caregivers performing skin to skin/breastfeeding/continuing work with KMC in the facility.

- How can we overcome these barriers, what are your suggestion?

10. During providing KMC if the newborn get sick how do you identify these babys and how do you manage them? (sending the baby for special care)
11. When do you **discharge** the KMC baby? Which criteria do you follow for discharge?
12. How do you motivate the caregivers to continue in the facility if they want to be discharged by their own decision?
13. How do you motivate the caregiver to continue KMC at home after discharge?
14. Can you give an example of a successful story?
15. Anything else to address?

Thank you for your participation!
